# Mercury exposure, malaria, and serum antinuclear/antinucleolar antibodies in amazon populations in Brazil: a cross-sectional study

**DOI:** 10.1186/1476-069X-3-11

**Published:** 2004-11-02

**Authors:** Ines A Silva, Jennifer F Nyland, Andrew Gorman, Andre Perisse, Ana Maria Ventura, Elizabeth CO Santos, Jose M de Souza, CL Burek, Noel R Rose, Ellen K Silbergeld

**Affiliations:** 1 The Johns Hopkins University Bloomberg School of Public Health, 615 N. Wolfe Street, Room E6642, Baltimore, Maryland, 21201 USA; 2 Department of Epidemiology, University of Maryland Medical School, Baltimore, Maryland, USA; 3 Institute Evandro Chagas (IEC), Fundaçao Nacional da Saúde, Belem do Pará-66090, Brazil

## Abstract

**Background:**

Mercury is an immunotoxic metal that induces autoimmune disease in rodents. Highly susceptible mouse strains such as SJL/N, A.SW, B10.S (H-2^s^) develop multiple autoimmune manifestations after exposure to inorganic mercury, including lymphoproliferation, elevated levels of autoantibodies, overproduction of IgG and IgE, and circulating immune complexes in kidney and vasculature. A few studies have examined relationships between mercury exposures and adverse immunological reactions in humans, but there is little evidence of mercury-associated autoimmunity in humans.

**Methods:**

To test the immunotoxic effects of mercury in humans, we studied communities in Amazonian Brazil with well-characterized exposures to mercury. Information was collected on diet, mercury exposures, demographic data, and medical history. Antinuclear and antinucleolar autoantibodies (ANA and ANoA) were measured by indirect immunofluorescence. Anti-fibrillarin autoantibodies (AFA) were measured by immunoblotting.

**Results:**

In a gold mining site, there was a high prevalence of ANA and ANoA: 40.8% with detectable ANoA at ≥1:10 serum dilution, and 54.1% with detectable ANA (of which 15% had also detectable ANoA). In a riverine town, where the population is exposed to methylmercury by fish consumption, both prevalence and levels of autoantibodies were lower: 18% with detectable ANoA and 10.7% with detectable ANA. In a reference site with lower mercury exposures, both prevalence and levels of autoantibodies were much lower: only 2.0% detectable ANoA, and only 7.1% with detectable ANA. In the gold mining population, we also examined serum for AFA in those subjects with detectable ANoA (≥1:10). There was no evidence for mercury induction of this autoantibody.

**Conclusions:**

This is the first study to report immunologic changes, indicative of autoimmune dysfunction in persons exposed to mercury, which may also reflect interactions with infectious disease and other factors.

## Background

Mercury has been recognized as a significant environmental and public health problem for more than 40 years, primarily for its effects on the developing nervous system, as expressed in tragic episodes of human poisoning in Japan and Iraq [1]. Awareness of the effects of mercury on the immune system has increased in the last decade [2,3].

In rodent models exposure to inorganic and organic mercury has a range of immunotoxic effects, functionally associated with decreased cell-mediated immunity and the induction of autoimmunity [4]. These effects vary with strain [5-7]. Both inorganic and organic forms of mercury are immunotoxic, although they differ quantitatively and qualitatively in their effects on the immune system; methylmercury may require metabolism into inorganic species to induce immunotoxic effects, such that the effects of methylmercury are delayed and reduced in appearance [6]. Ethylmercury (C_2_H_5_Hg^+^), the active compound in thimerosal and other medical compounds, induces in a dose-dependent pattern all the features of systemic autoimmunity that have been described after exposure to mercuric chloride (HgCl_2_) [8]. Mercury can enter the body through inhalation, as elemental mercury (Hg^0^), through dermal or eye contact, as ethylmercury, and by absorption through the gastrointestinal track, primarily as methylmercury (CH_3_Hg^+^) through ingestion of contaminated fish [1]. Inhaled Hg^0 ^vapor easily crosses the pulmonary alveolar membranes to enter the circulatory system, where it is primarily bound to red blood cells, and is rapidly distributed to the central nervous system, and the kidneys [9]. Mercury absorbed through skin contact is oxidized in the liver to Hg^2+ ^by glutathione [10]. After entering the blood stream, mercury is distributed to all tissues, including the brain, kidney, lungs, hair, nails, liver, fetus, milk, etc [1,10].

In the literature, no cases of frank autoimmune disease have been reported in persons exposed to mercury, occupationally or environmentally [3]. A few studies have examined relationships between mercury exposures and adverse immunological reactions, particularly in connection with mercury amalgam, but these are controversial [1]. At relatively high levels of occupational exposure, changes in immunoglobulins have been reported, but not consistently [3,11-13]. Nephropathy described in workers with either acute or chronic exposures to Hg^0 ^vapor may involve deposition of autoantibodies to basement membrane proteins in the glomerulus [3,14]. In a study of chloralkali workers, circulating anti-laminin antibodies were found in some workers as well as autoantibodies against glomerular basement membrane and circulating immune complexes, but no significant increases in antinuclear autoantibodies (ANA) were found [12]. No studies of immune parameters have been conducted in the large longitudinal studies of children exposed to methylmercury via fish consumption in the Seychelles or in the Faeroe Islands [1,15,16]. In a cross-sectional study of a maritime population of children with exposure to polychlorinated biphenyls and methylmercury via seafood consumption, numbers of naïve T-cell subsets (CD4^+^CD45RA), T-cell proliferation, and plasma IgM were decreased, while IgG levels were increased, relative to controls [17].

The goal of this study was to test the hypothesis that exposures to methylmercury and/or inorganic mercury may have effects on specific markers of mercury-induced autoimmunity, that is, ANA and antinucleolar (ANoA) autoantibodies, and in a subset of subjects anti-fibrillarin (AFA) autoantibodies. ANoA autoantibodies, a marker found in some human autoimmune diseases [18], have been reported to be elevated by mercury in mice [19]. More recently, Pollard et al. have proposed that ANoA antibodies targeting the nucleolar 34-KDa protein fibrillarin may be specific biomarkers of mercury-induced immunotoxicity [20,21]. Mercury-induced ANoA in mice reacts with a conserved epitope of fibrillarin [20,21], which is indistinguishable from the AFA response seen in scleroderma. A recent case-control study reported that severely affected scleroderma patients with AFA were more likely to have higher levels of mercury in urine, as compared either to less severely affected cases without AFA, or controls, suggesting an etiologic role for mercury in this autoimmune disease [22]. However, the sample size was small and levels of mercury were low in all subjects.

We were able to conduct this study in collaboration with an ongoing epidemiological surveillance of mercury exposures in Amazonian Brazil, where populations are exposed to both inorganic and organic mercury associated with gold mining activities [23,24]. In Amazonian Brazil, as in many other regions of the world, elemental mercury is used in liquid form for amalgamation of gold particles in placer deposits [23,25]. The gold miners are directly exposed to inorganic mercury and residents of downstream communities are exposed to methylmercury via consumption of fish. Extensive work has been done on many of these populations, documenting a range of exposures among miners and fish consumers [24-26], many well above the levels found in populations in North America and Europe, and well in excess of the levels found in the Seychelles and Faeroes cohorts [1], although lower than those reported in Minamata [27].

In this study we analyzed autoantibodies and mercury exposures in three populations from the state of Pará, Brazil. These groups were exposed to different types of mercury in different settings, with exposure to other risk factors not all of which were determined. Therefore, these may contribute to the observed differences among communities, in addition to mercury exposures. We report here that exposures to mercury are associated with significant increases in the prevalence of elevated serum ANoA.

## Methods

To test our hypothesis we examined three separate populations, selected from ongoing studies of mercury exposures and health status being conducted by FUNASA (Fundaçao Nacional de Saúde), under the leadership of Dr Santos of the Evandro Chagas Institute. The communities in our study were chosen from this surveillance database on the basis of differences in exposures to mercury and other risk factors in Pará, Brazil. At *Rio-Rato*, a *garimpo *or gold mine site, most of the population was directly involved in gold extraction and refining, resulting in relatively high but often episodic exposures to inorganic mercury, similar to those described by us and others [25,26]. This site is in the lower Tapajós River watershed, an area of high malaria transmission [28]. At *Jacareacanga*, a riverine community on the Tapajós River several hundred km downstream from the region of active gold mining in Pará, the inhabitants consume fish known to be contaminated with methylmercury [24,29,30]. There is little autochthonous malaria in this town but many people have histories of malaria because of contact with the nearby region [31]. Finally, at the village of *Tabatinga*, located on the lower Amazon River east of the Tapajós, the population has no direct or indirect contact with gold mining, and fish collected in this village have levels of methylmercury [24] within the guidelines for safe consumption recommended by the WHO and the US FDA [1]. Tabatinga has had no prevalent malaria over the past ten years, according to data from FUNASA (personal communication JM Souza).

### Study design

The overall design of the mercury surveillance studies conducted by Dr Santos is a community based, cross sectional survey of Brazilian populations in Amazonia, focused on the states of Pará, Amazonas, Acre, and Rondônia, including gold mining sites, riverine communities, and populations without exposure to mercury. The study design is described in detail by Santos and colleagues [29,32]. In all studies, a census was first conducted at each site to determine sampling strategy. Subjects were then contacted by house-to-house survey and enrolled in proportion to the population in terms of age and gender. Overall, between 80 and 90% of contacted persons consented to participate at each site. Information was collected by interview, administered in Portuguese by trained personnel, to provide information on demographics (age, gender, educational attainment), diet (with particular emphasis on fish), birthplace, current/previous occupation (including use of mercury), income, health status, reproductive history (women), drug and alcohol use, past/current malaria, number of people per household, time residing at the site, and medical history. A short clinical examination was conducted, and samples of hair, blood, urine, and stool were taken for laboratory analyses, including mercury levels in hair and urine. Malaria was assessed by questionnaire to determine past history of malaria (self reported), as well as by thick smears taken to determine prevalent malaria. All smears were read by trained technicians. Data on past malaria were stratified using Baird as reference [33], in which he determined the minimum number (4) of prior malaria infections associated with acquisition of functional immunity (i.e., no disease and/or parasitemia after biting). The study was approved by the institutional review board of the IEC and FUNASA. The University of Maryland Medical School and the Johns Hopkins Medical Institutions Institutional Review Boards also approved the analyses conducted in this study.

### Mercury exposure

Subjects' exposure to mercury was determined in two ways. First, information was gathered by questionnaire on occupational history (contact with and use of mercury in gold mining), and/or fish consumption (by weekly frequency and predominant types of fish consumed). Second, mercury concentrations were measured in biologic compartments. For persons in Tabatinga and Jacareacanga with chronic exposure via fish consumption, hair mercury (μg Hg/g of hair) was used as the exposure biomarker as recommended [1]. Hair samples were collected in 2 cm lengths (from the scalp) and analyzed using standard methods of atomic absorption spectrophotometry by cold vapor technique in the laboratory of Dr Santos, which participates in the international QA/QC program with the Université de Quebec [34]. For persons with occupational exposures to inorganic mercury, in Rio-Rato, urine mercury was used as the biomarker (μg Hg/L of urine, no data on creatinine was available). This is the standard method utilized by Santos and others for assessing occupational exposures to inorganic mercury and generally reflects relatively recent exposures [1,35].

### Immunologic outcomes

Blood samples were collected by venipuncture and sera were separated on site by centrifuge, aliquoted and immediately frozen on liquid nitrogen for transport by air to the IEC in Belem (Pará). Aliquots of frozen serum were stored at -80°C and then transferred on dry ice to Baltimore via air transport accompanied by Dr Silbergeld. All analyses were done under blinded conditions.

### Detection of ANA/ANoA

The serum samples were stored at -80°C until analysis. Each aliquot was thawed and 10 μL taken for analysis by indirect immunofluorescence (IIF) microscopy using commercially available slides prepared from human epithelial cells (HEp-2) as substrate (INOVA Diagnostics) following the methods of Burek and Rose [36]. The slides were stored in the dark at 4°C until they were analyzed by a blinded reader (IAS or AG). Randomly selected slides were re-checked by an experienced immunologist (CLB).

### Detection of AFA

The antigen proteins were obtained from rat liver nuclei [20], and the proteins were separated by 15% SDS-PAGE. Preparations were first fractionated by SDS-PAGE and subsequently transferred to nitrocellulose and immunoblotted. Briefly, nitrocellulose was blocked in PBS/0.1% Tween-20/5% dry milk for 2 h at room temperature. Incubation with primary antibody (serum samples) (1/50 in blocking solution) was performed at room temperature for 1 h, followed by 3 washing steps of 10 min each in PBS/0.1% Tween-20. Secondary antibody (horseradish peroxidase-conjugated goat anti-human IgG) (Caltag Lab, CA) was used at a dilution of 1/2000 in blocking solution for 1 h at room temperature followed by 3 washes of 10 min each in PBS/0.1% Tween-20. Bound antibody was detected using chemiluminescence. The 34 KDa protein was detected by molecular weight using serum of scleroderma (SC) patients as a positive standard. The SC serum revealed one band at the expected molecular weight of 34 KDa.

### Data analyses

The concentration of serum autoantibodies is expressed in terms of the dilution factor at which fluorescence could still be detected. Detection of autoantibodies at a serum dilution of ≥1:40 is considered "positive" for most clinical uses [37]. However, detectability at dilutions between 1:10 and 1:40 can also have health implications [37] and may be relevant as biomarkers of mercury exposure. Since we are studying the autoantibodies as biomarkers of immunotoxicity rather than as indicators of disease, we present our findings at both dilutions, ≥1:40 and ≥1:10.

### Statistical analysis

Means for continuous variables (median for variables with skewed distributions) and percents for categorical variables were computed for the descriptive analysis in our data. Chi Square test was used to compare categorical variables and Student's t-test was used for continuous variables. In Jacareacanga we stratified hair mercury levels based on World Health Organization guidance (≤8 or ≥8 μg/g hair). In Tabatinga we stratified exposure by the observed median level (5.57 μg/g) since most hair mercury concentrations were below 8 μg/g. In Rio-Rato we used urine mercury levels based on WHO guidance (≤5 or ≥5 μg/L). We used the mean age for each population to stratify by age. Logistic regression modeling was used to evaluate the effect of mercury exposures on prevalence of ANA and ANoA (for 1:10 and 1:40 cutoffs), while controlling for age, sex, prevalent malaria, past history of malaria, and occupation. All data were analyzed using the SAS v.8.1 statistical package.

## Results

Because of substantial differences among the populations and sites, we present the results for each site separately.

### Tabatinga

**Figure 1:**
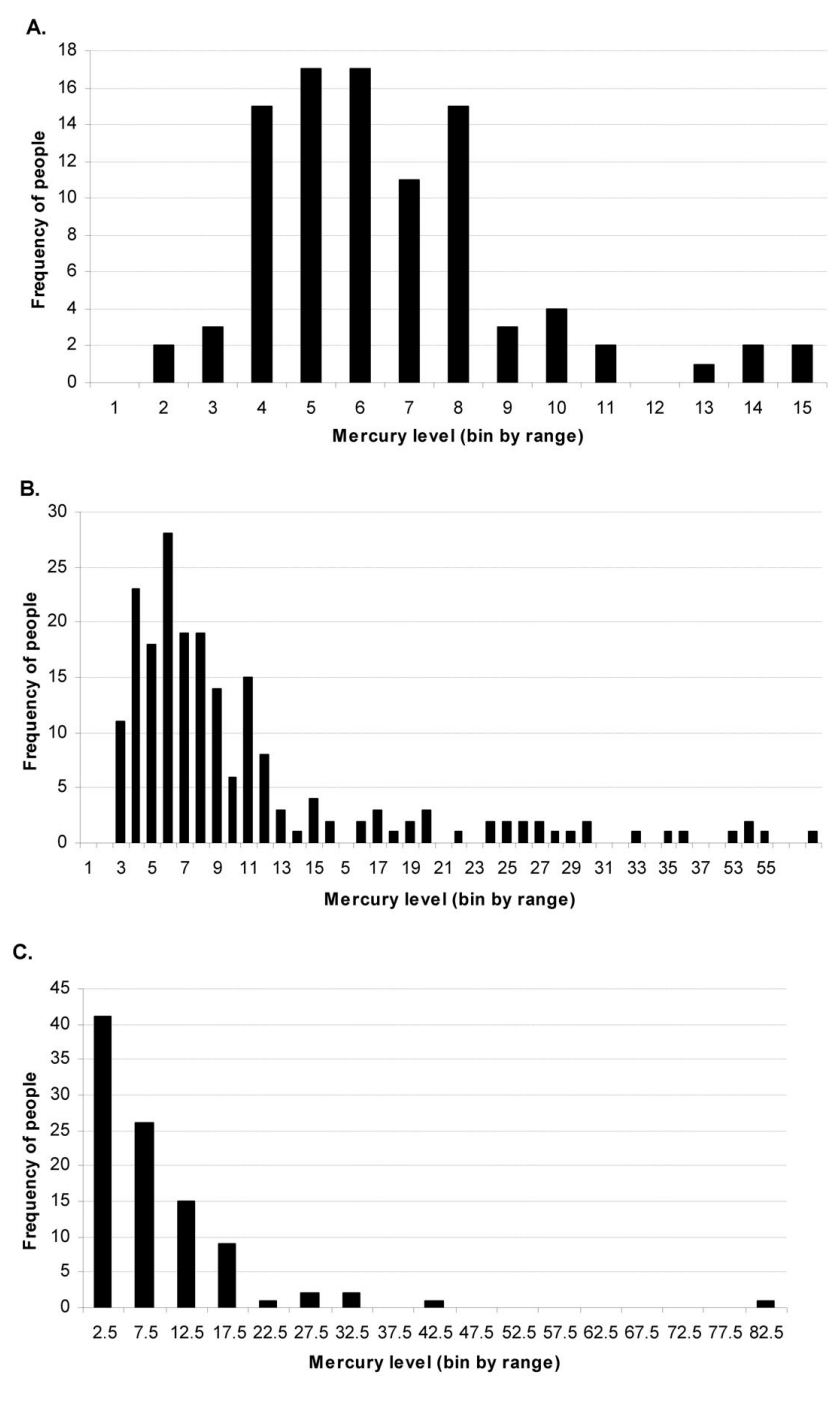
**Distribution of mercury levels **Population distributions are shown for (A) Tabatinga and (B) Jacareacanga in μg Hg/g hair and (C) Rio-Rato in μg Hg/L urine.

Tabatinga is a typical riverine community in the lower Amazon. The community sample consisted of 98 adults, with 73% females, and a mean age of 44 years, (Table 1). This community has no occupational exposures to inorganic mercury, and the fish consumed have relatively low methylmercury contamination. The distribution of hair mercury is shown in Figure 1A; the majority of the persons had hair mercury levels below 8 μg/g. The median hair mercury concentration of 5.57 μg/g is higher than that reported in European and North America populations, which may reflect the very high frequency of fish consumption rather than excessive fish contamination [38]. No present malaria cases were found, and only 10% reported any past malaria (Table 2). Otherwise, the population was in good health.

**Table 1:** Demographic characterization of the 3 populations

				Current Occupation (%)				Prev. Occupation
				
Populations	N	Age [Mean]	Sex (%) F/M	Gold Mine	Fisherman	Others	Students	Gold Miner (%)
Tabatinga-adults	98	44	73/27	0	1.1	98.9	0	0
Jacareacanga	140	25	54/46	0	2.2	72.4	25.4	9.4
Rio-Rato	98	30	35/65	54	0	46	0	N/A

N/A = data not available from original survey.

**Table 2:** Malaria and Hg data from the 3 populations Malaria status (prevalent and reported past infections) and mercury exposures in the three populations.

	Malaria		Hg (median)		Hg
	
Populations	Prevalent (%)	History (%)	Urine (microgram/L)	Hair (microgram/g)	range values
Tabatinga-adults N = 98	0	10.1	ND	6.4	1.19–16.96
Jacareacanga N = 140	0	69.6	ND	8	0.29–58.47
Rio-Rato N = 98	93.9	N/A	4	ND	0.01–81.37

N/A = data not available from original survey.

ND = analysis not completed in original survey.

**Figure 2:**
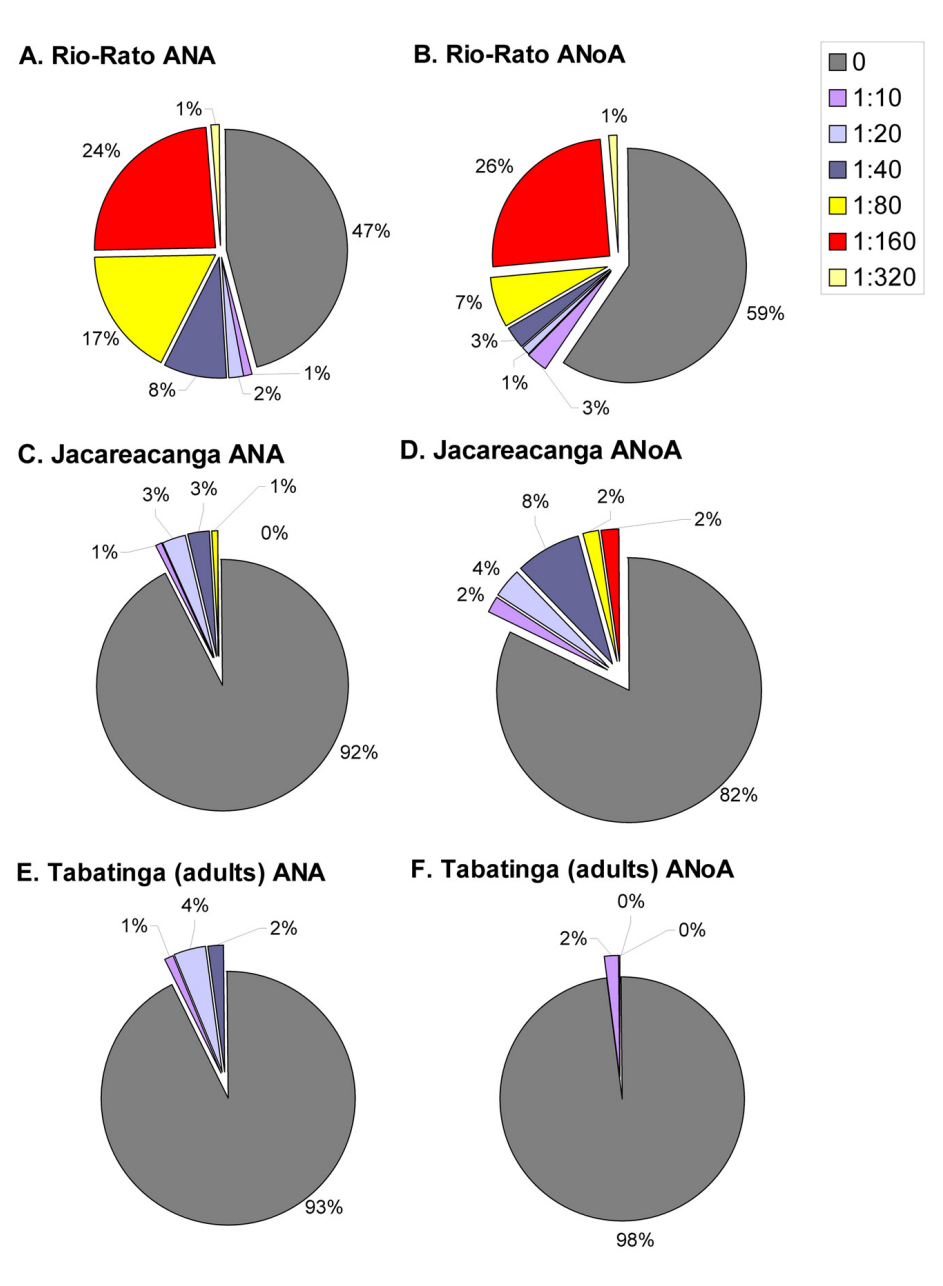
**Detectable levels of serum autoantibodies **Population distributions of (A, C, E) ANA and (B, D, F) ANoA are shown for (A&B) Rio-Rato, (C&D) Jacareacanga and (E&F) Tabatinga, at varying serum dilutions.

The prevalence of detectable ANA and ANoA in the Tabatinga samples was very low (Table 3; Figure 2). Most measurements (90%) were not detectable even at the lowest (1:10) dilution. These data are similar to those recently reported for a referent population in Sao Paulo [39]. In the few subjects with ANA or ANoA detectable at ≥1:10, there was no relationship between ANA or ANoA for any of the variables studied.

**Table 3:** Percentages of detectable ANA and ANoA in serum from the 3 populations

	ANA (%)			ANoA (%)			ANA + ANoA (%)		
Populations	<det	≥1:10	≥1:40	<det	≥1:10	≥1:40	<det	≥1:10	≥1:40

Tabatinga-adults N = 98	92.9	7.1	2.0	97.9	2.1	0	100	0	0
Jacareacanga N = 140	89.3	10.7	3.6	82.0	18.0	13.0	100	0	0
Rio-Rato N = 98	45.9	54.1	51.0	59.2	40.8	36.7	89.0	11.0	10.0

<det = below detection level at lowest dilution.

ANA ≥1:10 or ANoA ≥1:10 percentages include ANA 1 1:40 or ANoA ≥1:40 percentages.

### Jacareacanga

*Jacareacanga *is a riverine settlement of approximately 500 persons, located on the mid-Tapajós River. The 140 subjects consisted of 54% women and had a mean age of 25 years (Table 1). Fish are the primary protein source and piscivorous species sold at local markets have reported to have elevated concentrations of methylmercury [29,30]. No persons reported current employment in gold mining or refining, but some persons reported a history of such activities. The distribution of hair mercury is shown in Figure 1B. Median hair mercury levels were 8 μg/g (Table 2), substantially higher than that found in unexposed populations [1]. Fish consumption was the major predictor of hair mercury; previous occupation as a gold miner was also related to higher hair mercury concentrations. No subjects were positive for malaria by blood smear at the time of survey (Table 2). However, a majority reported a history of past malaria (Table 2). Among these subjects, 50% reported 2 or fewer infections, while the maximum number of past infections reported was 6.

As shown in Table 3, nearly 11% of the population had detectable ANA ≥1:10, and nearly 20% had detectable ANoA ≥1:10. In those subjects where ANA was detectable, most (96.4%) presented at low concentrations, while 13% had ANoA detectable at 1:40 (Figure 2). No subjects were positive for both autoantibodies. A significantly higher percentage of subjects with detectable ANoA (33%) had hair mercury levels greater than the median value of 8 μg/g. In the logistical model only mercury, from all the variables studied, was significantly correlated with the presence of ANoA (≥1:10) (Table 4). Individuals with higher hair mercury levels, who reported any past malaria, were more likely to have detectable concentrations of ANoA (40%) as compared to those with low mercury levels. In persons reporting fewer than 4 past malaria infections, hair mercury was positively correlated with the presence of detectable ANA (≥1:10; ≥1:40) and ANoA (≥1:10) (Table 5). In persons with low hair mercury, there was a positive correlation of number of past malaria infection with detectable ANA at either ≥1:10 or ≥1:40.

**Table 4:** Jacareacanga-odds ratio between risk factors and prevalence of ANoA ≥1:10 Logistical model for odds of detectable ANoA (≥1:10) and mercury exposure, gender, age, occupation, and malaria history, p < 0.05*.

Variable	Odds ratio	95% Confidence interval	p-value
Hg	3.27	1.28 – 8.37	0.014*
Gender	1.16	0.44 – 3.02	0.769
Age	0.93	0.36 – 2.39	0.871
Past-malaria	1.28	0.43 – 3.83	0.663
N past-malaria infections	1.18	0.39 – 3.55	0.772
Other occupations: gold miner	0.74	0.14 – 3.75	0.711

**Table 5:** Serum ANA and ANoA (Jacareacanga) stratified for mercury and past malaria infections P values obtained comparing Hg <8 with >8 μg/g hair (* p < 0.05) and number of malaria infections <4 with ≥4 (^§^p < 0.05).

	**# malaria infections <4 (%)**	**# malaria infections ≥4 (%)**
**Hg >8:**		
ANA 1:10	3.61	17.65 ^§^
ANA 1:40	0	11.76 ^§^
ANoA 1:10	12.05	17.65
ANoA 1:40	8.43	17.65
**Hg ≥8:**		
ANA 1:10	14.81 *	10.00
ANA 1:40	11.11 *	0
ANoA 1:10	33.33 *	30.00
ANoA 1:40	22.22	20.00

### Rio-Rato

*Rio-Rato *is a gold mining community, where a small settlement has grown up around a still active mining site in the mid-Tapajós watershed. Approximately 2/3 of the population was male with a mean age of 30 years (Table 1). Educational and socioeconomic variables were low. Urine mercury levels (4 μg/L) were lower than those found in other mining populations in Amazonia [25,26,40] (Figure 1C). Only 6 had levels ≥25 μg/L, the median value found by us in another gold mine population [40]. This may have been due to the timing of our visit, during the dry season, when gold amalgamation activities were reduced. A high degree of variability in urine mercury levels among gold miners has been reported by others [26,41]. Because of this, we used exposure history to characterize mercury exposures in this population.

This region has a high rate of malaria transmission [28,31]. Over 90% of the Rio-Rato population had prevalent malaria, detected by thick film slide at the time of the survey (Table 2). No data on past malaria episodes were collected. Over half of the population had ANA detectable at ≥1:10, and nearly half had ANoA detectable at ≥1:10 (Table 3; Figure 2). At or above 1:40, the Rio-Rato population still presented with a high prevalence of elevated ANA (51%) and ANoA (36.7%) (Table 3; Figure 2). In 10% of the population, levels of both autoantibodies were detectable at 1:40. About a quarter had concentrations up to a dilution 1:160 and some persons in this sample had very high concentrations of autoantibodies, detectable up to a dilution of 1:320. The likelihood of ANoA detectable at 1:40 was significantly higher in those individuals with a longer history of work in gold mining (≥7 years compared to <4 years).

**Figure 3:**
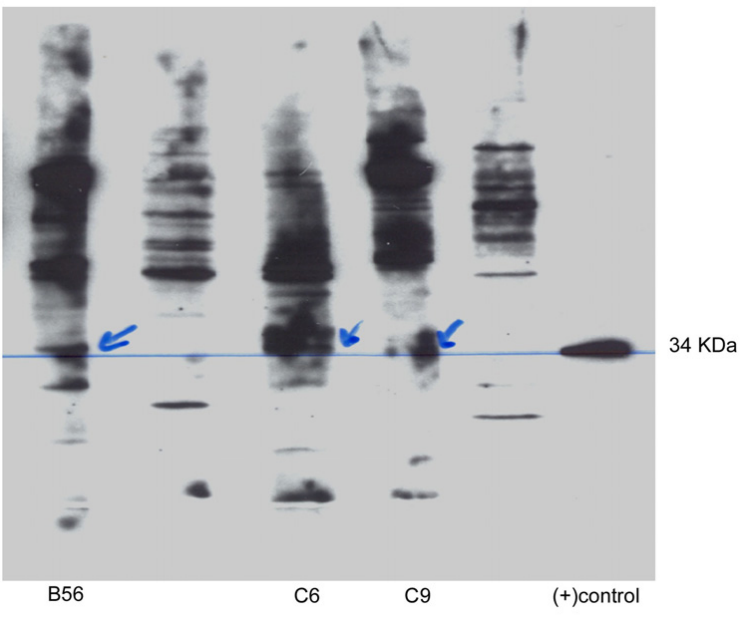
**AFA in Rio-Rato serum samples **Photograph of denaturing gel electrophoresis of 3 AFA positive samples from 41 ANoA positive serum samples previously determined by IIF. Fibrillarin = 34 Kda protein.

The presence of autoantibodies against the 34 KDa protein fibrillarin was determined by immunoblotting in those serum samples from Rio-Rato with ANoA detectable ≥1:10. Of 40 subjects, 3 had serum with detectable AFA, as shown in Figure 3.

## Discussion

In this paper we report the first data on specific biomarkers of autoimmune dysfunction in persons exposed to inorganic mercury or methylmercury. One earlier study reported elevations in anti-laminin autoantibodies in workers exposed to mercury [12]; however, no correlation with mercury exposure was observed. While our data are limited by sample size, and are likely influenced by other variables in addition to mercury, the results are consistent with the experimental literature indicating that mercury can alter immune function and increase circulating levels of autoantibodies, including ANA and ANoA [5,6,42].

There was an overall qualitative correlation between mercury exposures and levels of ANA or ANoA, both by study site and within study sites. Our ability to compare these populations more directly was limited by differences in the original study design with respect to mercury exposure assessment (hair at Jacareacanga and Tabatinga, urine at Rio-Rato). Persons from Tabatinga, with the lowest range of mercury exposures, had lower prevalence of detectable ANA or ANoA, and in those few persons with detectable autoantibodies, the concentrations were low. Nonetheless, Tabatinga subjects had significantly elevated mercury levels, as compared to North America populations [1,38], which is probably attributable to their very high intake of locally caught fish, such that even though these fish had methylmercury levels below the US FDA or WHO guidance, these consumption rates resulted in elevated body burdens, as compared to North Americans eating fish much less frequently [43]. Persons from Jacareacanga were exposed to methylmercury from fish consumption. The median hair mercury levels in Tabatinga and Jacareacanga are relatively close, but the distribution of hair mercury in Jacareacanga shows that there are many persons with exposures well above those obtained in Tabatinga. In Jacareacanga subjects, higher prevalence of detectable ANoA was observed, mostly at low concentrations (1:10 or 1:40), but several had levels measurable in dilutions as high as 1:160. In Rio-Rato persons were highly exposed to inorganic mercury from gold mining activities, as well as methylmercury via fish consumption. Detectable levels of ANA and/or ANoA were prevalent and detectable at high concentrations (1:320).

It is possible that exposure to inorganic mercury may be more "autoimmunogenic" than exposure to methylmercury, as shown in mice models [6]. In contrast to studies in mice [20,21], we found little evidence that AFA, levels are specifically affected by mercury. This may indicate a difference between humans and mice. However, as shown in Figure 3, there appear to be many unidentified nuclear antigens observed in serum samples from this population, which were not observed in either the SC serum, or in studies of US control subjects (data not shown). It would be very pertinent to analyze sera from all these three populations, using a range of other nuclear antigens known to be targeted in autoimmune diseases [19,37].

We examined the serum ANA and ANoA results at both 1:10 and 1:40 dilutions. The results in Jacareacanga and Rio-Rato subjects are clearly different from studies of healthy individuals in the US and Brazil [39]. Interpretation must be cautious. Tan et al. [37] showed that many "healthy individuals" (31.7%) show detectable ANA at dilutions <1:40. This suggests that such a cutoff point for serum dilution may have relatively little diagnostic value. However, the purpose of this study was not to detect persons with latent autoimmune disease, but rather to use these antibody measurements as biomarkers to test the hypothesis that mercury exposures might induce autoimmune dysfunction. A recent publication on the prevalence of ANA in serum of normal blood donors in Brazil found no age-related differences in prevalence of detectable ANA among adults, and that very few subjects had detectable ANA at dilutions >1:40 [39]. None of the subjects in these populations were reported to have autoimmune disease or overt clinical disease of any type, except malaria, but only routine clinical assessments were done.

In Rio-Rato and Jacareacanga subjects, other risk factors were related to elevations in ANoA and ANA. In Rio-Rato, time spent at the site and in gold mining was positively correlated with likelihood of elevated ANoA. This variable, time spent at the site, may represent length of exposure to both mercury and malaria infection. In Jacareacanga, there was a positive relationship between malaria (any past reported cases) and likelihood of elevated ANoA. We examined these potential biological interactions among mercury, malaria, and autoimmune biomarkers further, because of studies demonstrating that repeated malaria infections are associated with increased levels of autoantibodies, including ANA, presumably due to cytotoxic damage and exposure of intracellular epitopes [44,45]. Other studies have shown that autoantibodies are produced in mice infected with malaria, which react with several nuclear antigens, namely RNA, soluble nuclear material and DNA [46]. Our data indicate that in persons with lower mercury exposures (less than the median of 8 μg/g hair), increasing number of past malaria infections (≥4) were associated with increased likelihood of ANA, but not ANoA. In persons with higher mercury exposure, increased malaria exposure did not further increase ANA. We do not, at present, have an explanation for these observations, except to speculate that higher mercury exposures may induce a strong autoimmune dysfunction, such that additional effects of malaria are not significant.

It is difficult to draw any firm conclusions from these analyses, since the malaria data in Jacareacanga were based upon unconfirmed self-reports. We could not test this hypothesis in the Rio-Rato group, since almost all subjects had prevalent malaria and extensive histories of past infection.

We have reported a suggested correlation between mercury exposures and number of past malaria infections among gold miners in another gold mine settlement, in Brazil, at Piranha [40]. We have also reported that exposure of mice to low levels of mercury both decreases host resistance to murine malaria (*Plasmodium yoelii*) and impairs acquisition of immunity to murine malaria in the Nussenzweig model [47]. Mercury may reset immunologic responses to malaria, to increase expression of autoantibodies through its documented effects to up regulate Th2 mediated immune responses [48,49].

Finally, we may speculate as to why we have been able to observe associations between mercury exposures and these biomarkers of autoimmune dysfunction in these populations, while most clinical or epidemiological studies of mercury and immunotoxicity have been negative or only weakly positive [11-13]. In these other studies, the cohorts were relatively small (between 44–70), and they were exposed only to elemental or inorganic mercury through working in mercury or chloralkali plants [11,13]. No reports of exposure to other risk factors, such as infectious diseases, were reported. In our study, some of the exposures were chronic in nature and included exposures to methylmercury (certainly for fish consumption in Amazonian populations, where fish form the major portion of the protein consumed) [30,50]. In contrast, the gold miners were likely to have relatively variable exposures to inorganic mercury, with episodes of very high inhalation exposure [25,26,41]. The role of genotype may also be important. In rodents, genotype clearly plays an important role in both modulating the immune response to inorganic mercury as well as toxicokinetics [5-7,51]. In susceptible mice, induction of genetically restrictive ANoA by mercury are linked to mouse MHC (H-2) haplotype s and q [7], while most other haplotypes confer relative resistance [52]. Non-MHC genes decide the strength of ANoA response in susceptible mice exposed to mercury [6,7]. In addition, mercury toxicokinetics differ among inbred mouse strains. As Nielson and Hultman [52] demonstrated, there is a correlation between mercury toxicokinetics and AFA production in mice. In this study, the populations in these communities represent a wide range of ethnicities, including Europeans, Africans, and indigenous groups of Amazonia. Their immunogenetics may include persons with increased susceptibility to mercury-induced autoimmune dysfunction.

## Conclusions

Our study is the first to report a correlation between biomarkers (ANA and ANoA) and mercury exposure in humans. In addition, co-exposures to mercury and infectious diseases, including malaria, may set the stage for eliciting discernible alterations in immune function. Whether such co-exposures increase the risks of autoimmune disease will require further studies, which are underway.

## List of abbreviations

ANA – antinuclear autoantibodies

ANoA – antinucleolar autoantibodies

AFA – anti-fibrillarin autoantibodies

IIF – indirect immunofluorescence

## Competing interests

The authors declare that they have no competing interests.

## Authors' contributions

IA Silva carried out the analysis of autoantibodies, participated in the statistical analysis, and drafted the manuscript. JF Nyland helped in autoantibody analysis and technical editing of the manuscript; A Gorman helped in autoantibody analysis; AM Ventura, JM de Sousa, and ECO Santos carried out the field studies, malaria assessments, and mercury analysis. CL Burek and NR Rose guided the antibody analysis. EK Silbergeld participated in the design and coordinated the study; she also participated in the Jacareacanga field study, and in the draft of the manuscript. All authors read and approved the final manuscript.

## Pre-publication history

The pre-publication history for this paper can be accessed here:


